# Through the Eyes of Black Nurses: The Impact of the Nurse Training Act of 1964

**DOI:** 10.1177/15271544241296825

**Published:** 2025-01-03

**Authors:** Hafeeza Anchrum

**Affiliations:** 1 Department of Africana Studies, 142839 Program on Race, Science and Society, University of Pennsylvania, Philadelphia, Pennsylvania, United States

**Keywords:** nursing schools, federal government, nursing education, civil rights, social justice, racial segregation

## Abstract

The Nurse Training Act (NTA) was passed by the United States Congress in 1964 in response to growing national concerns about a shortage of nurses. The legislation provided substantial funding for nursing education to increase the supply of nurses and improve the quality of nursing services. However, contemporary investigations into the causes of the shortage focused on the professional lives and experiences of white women, who were the main target of nurse recruitment and retention efforts. This research shifts the focus to Black women nurses, examining both the causes of the shortage and the impact of the NTA on the nursing workforce from their perspective. It argues that a key achievement of the NTA—alongside the Civil Rights Act of 1964—was the dismantling of legalized racial segregation in professional nursing schools, a major barrier to the development of the Black professional nursing class. Yet, this federal intervention was not simply bestowed; it was the result of decades of sustained advocacy by Black nurses from the 1890s to the 1960s to secure equal educational opportunity and federal support for their civil liberties. Viewing this landmark legislation through the eyes of Black nurses underscores the federal government's pivotal role in both promoting and obstructing racial and healthcare equality. As the nation faces yet another nursing shortage, coupled with the eradication of affirmative action and diversity, equity, and inclusion initiatives, this perspective is especially timely and important for informing current and future issues pertaining to health equity.

## Introduction

Ms. Marie Harvey's desire to pursue a career in nursing was motivated by her experience growing up in poverty and working in domestic service alongside her mother. “I was never really a person who thought, ‘Oh, nursing is what I want to do.’ But Mother said, ‘You got to do something. You are not going to work just as a servant in these white homes.’ So, I went to nursing school, so I didn’t have to do that type of work,” Harvey recalled (Marie Harvey, personal communication, August 1, 2016). For most of the twentieth century, the majority of Black^1^ women worked in agricultural and domestic service for meager wages. Discriminatory laws and practices, reinforced by racist stereotypes about Black womanhood put Black women at a disadvantage in the workforce compared to white women and men (Hill Collins, 2009; Nina, 2019; Roberts, 2011). Following her mother's advice, Harvey was intent on breaking free from these institutional constraints. She committed herself to her studies, and in 1951, she was accepted into the internationally renowned Philadelphia General Hospital Training School for Nurses. Harvey, however, experienced a humiliating turn of events when she visited the school to accept her admission offer. She described how the white Director of Nurses rescinded the offer upon learning that Harvey was Black:

She said, “Ah this is a mistake, oh my goodness, this is just a big mistake!” See, you didn’t have to put your race on the application. I guess they thought they could tell by the way you spoke [over the phone] whether you were black or not. She was just upset. She said, “You just sign this paper and say you won’t come here, that this was just a big mistake.” (Marie Harvey, personal communication, August 1, 2016)

Although there was a nation-wide shortage of nurses, in the 1950s, most nursing schools refused admission to Black students (Staupers, 1961; Thoms, 1929). Harvey had also applied to the nursing program at Philadelphia's Mercy-Douglass Hospital, the state's only Black-run nursing school. Harvey was admitted to Mercy-Douglass and earned her nursing diploma in 1954, a decade before the enactment of the Civil Rights Act and the Nurse Training Act (NTA), which together desegregated U.S. professional nursing schools (Anchrum, 2021). 
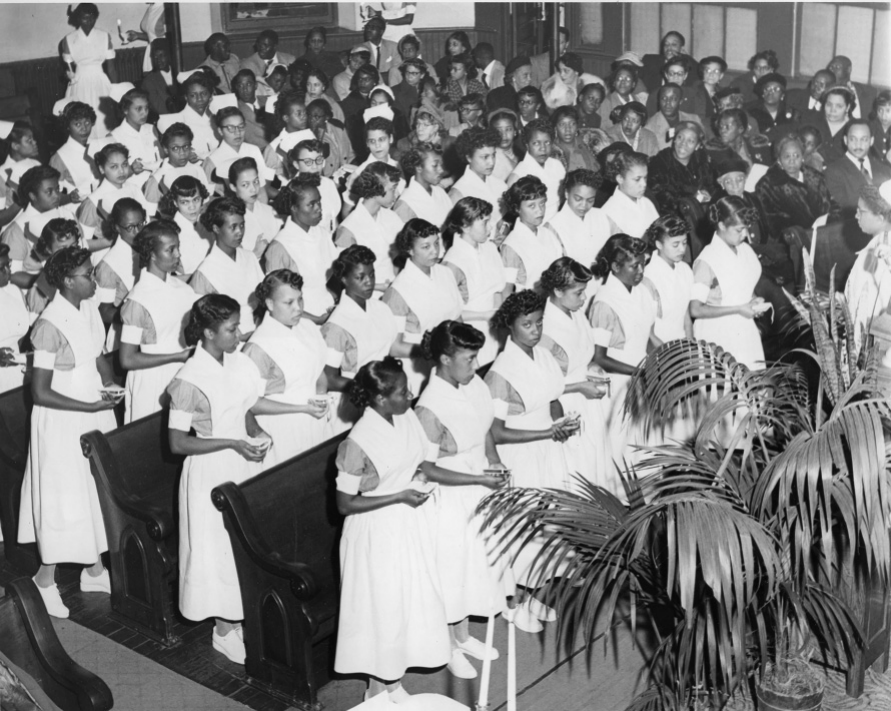


## Background

HR 11241, also known as the NTA, was passed by the United States Congress (USC) on September 4, 1964, in response to an acute and worsening national nursing shortage. Under Title VIII of the Public Health Service Act, the NTA allocated $238 million to nursing education with the aim to increase the supply of nurses (USC, 1964; USPHS, 1965). Research conducted from the 1930s to the 1960s identified different social, cultural, and economic factors driving the shortage, with one significant factor being an inadequate educational system (Goostray, 1941; “Nursing at Recent Hospital Conventions,” 1936; “Nursing at the AHA Convention,” 1938; United States Department of Labor, Bureau of Labor Statistics, 1947; United States Public Health Service, U.S. Department of Health, Education, and Welfare, 1963). The identified factors largely reflect the perspective and professional experience of white women, who were the primary focus of nurse recruitment and retention initiatives. Similarly, retrospective analyses of the twentieth-century nursing shortage overlook how the causes and effects of the shortage varied along racial lines (Keeling et al., 2017; Lynaugh, 2008). As a result, we do not have a full grasp of how the NTA contributed to reducing health, gender and racial inequities. While Black nurses faced similar challenges as white nurses owing to their shared gender and occupational identities, their professional lives were uniquely impacted by the interlocking systems of sexism, racism, and economic oppression. Therefore, this essay analyzes the causes of the shortage and in turn, the impact of the NTA on the development of the nursing workforce from the standpoint of Black women nurses. It argues that, in addition to investing in nursing education, the NTA transformed and strengthened the nursing workforce by abolishing legalized racial segregation in professional nursing schools, thereby eliminating a key structural barrier impeding the growth of the nurse supply. However, I assert that when viewed through the eyes of Black nurses, this progressive legislation was not freely given—it was fought for and won.

## Methodology

This article is the first extensive analysis of the NTA as a civil rights initiative, drawing on a broad array of primary and secondary sources from 1890 to 1970. Key primary sources include official publications from the U.S. Public Health Service, particularly from the Department of Health, Education, and Welfare's Division of Nursing, which offer critical insights into issues of nursing education and workforce needs that led to the NTA’s creation and subsequent evaluations. Congressional hearing reports further illuminate the NTA's objectives, execution, and later amendments. Original research explains the roots and evolution of the nursing shortage, highlighting its effects on nursing practice and public health. Descriptive studies by Black nurse pioneers, such as Mabel Staupers, Mary Elizabeth Carnegie, and Estelle Riddle Osborne were essential in uncovering how the causes and impact of the nursing shortage differed across Black and white communities, illuminating the need for federal action to address these disparities. Carnegie’s 1965 analysis of the impact of integration on nursing is particularly noteworthy as one of the few studies to discuss the NTA as a tool for advancing educational equality. These investigations were supported by oral histories of rank-and-file nurses that vividly illustrate the institutional barriers that Black women faced in pursuit of a nursing career. Moreover, secondary sources provide a comprehensive examination of the social and health context, detailing the significant racial disparities in access to healthcare and the chronic shortage of Black medical professionals. A small yet important body of research elucidates the structural barriers faced by Black nurses, chronicling their evolution from prioritizing self-help and institutional development to fighting for racial inclusion and equal rights. 

 Together, these sources reveal how, amidst rising concerns over a national nursing shortage, Black nurses skillfully connected this issue to the impact of racially discriminatory laws and practices. They argued that the causes of the shortage went beyond an inadequate educational system and recruitment of white women, emphasizing instead the role of structural racism and the exclusion of Black women. Their persistent advocacy, combined with mounting pressure from civil rights activists in the 1950s and 1960s demanding federal guarantees of equal protection and opportunity for all, set the stage for the passage of the NTA. Enacted two months before the 1964 NTA, Title VI of the Civil Rights Act of 1964 outlawed discrimination based on race, color, or national origin in programs that received federal aid (USC, 1964; USPHS, 1965). Therefore, nursing schools that were racially segregated or did not take active steps to integrate their student body were not eligible to receive NTA funds. This stipulation effectively desegregated the nation's professional nursing schools. Additional provisions and revisions to the Act mitigated other systemic barriers that impeded access to nursing education (United States Public Health Service, U.S. Department of Health, Education, and Welfare, Division of Nursing, Program Review Committee, 1967). The enactment of the NTA was another significant, though virtually unknown, civil rights victory for Black nurses in their quest to secure the full right to care.

Viewing the impact of the NTA through the eyes of Black nurses illuminates the paradoxical role of the federal government as both a catalyst for – and a barrier to – racial justice, underscoring the importance of sustained grassroots resistance. This perspective is especially relevant today, as the nation faces yet another nursing shortage amid dogged efforts to roll back civil rights gains.

## Discussion

### 1890s to World War II

Historically, the State has been instrumental in inhibiting the professional growth of Black nurses. At the turn of the twentieth century, deep racial hostility and political subordination of Black Americans was widespread. The ideology invented in the Enlightenment era that people of African descent were innately inferior to those of European descent was translated into social policy (Roberts, 2011). Local and state legislatures in the South enacted Jim Crow laws that enforced the separation of white people from people who were not racialized as white in nearly every aspect of daily life. In the North, an intricate system of discriminatory policies and practices also served to demarcate racial lines and prevent Black Americans from fully participating in society. The codification of racism was strengthened by the 1896 Supreme Court ruling in the case of *Plessy v. Ferguson*, which permitted the existence of “separate but equal” facilities (Kendi, 2016). Racist ideology justified, and racist laws enabled, the aspirations of elite white nurse leaders to create a formal system of nurse training exclusively for the social and economic benefit of white, middle-class, Protestant, and native-born women. The rigid standards defining who qualified as a trained nurse were reflected in the policies and practices of nursing schools and medical facilities nationwide. In the South, Black women were categorically denied entry to nursing schools and, in the North, they were excluded by way of a strict racial quota system. Racial segregation was also enforced in the employment, governance and organizational sectors of nursing (Carnegie, 2000; Hine, 1989). This race-based hierarchy of humanity, rooted in the dogma of white supremacy, established the unequal power relations between Black and white nurses and set in motion a near century-long struggle by Black nurses for the right to equal access and opportunity.

Black America's resistance to state repression and anti-Black racism was expressed through the creation of their own network of hospital nursing schools within the larger healthcare establishment. The objective was to cultivate a professional class of Black nurses to address the health needs of their communities while providing meaningful and higher-paying work for Black women. The combined effects of poverty, institutionalized racism, and gender discrimination on the liberties of Black women necessitated a communal effort to train and develop the Black nurse. Every sector of the Black community, from wealthier elites to poor citizens contributed to the establishment and continual operation of these schools, and in exchange, Black nurses dedicated their service to the community (Anchrum, 2021; Hine, 1989; Smith, 2010). Their endeavors reflected the principles of self-reliance, racial solidarity, and economic independence advocated by contemporary race leaders and embraced by the larger community as a mechanism for racial uplift (Gaines, 1996; Gamble, 1989). White philanthropists also contributed significantly to the movement to train Black nurses by establishing hospitals and nursing schools and funding educational initiatives. However, unlike those sponsored by the Black community, the schools established by white donors and municipal governments were segregated and generally overseen by white nurses. Such practices reflected the racist idea that Black people lacked the intelligence for leadership and self-governance. By the mid-1920s, there were approximately 40 Black nursing schools and 200 hospitals in the nation, most of which were established in southern states where the brutal system of Jim Crow racism and the large Black population created a greater need (Hine, 1989). Black nurses also formed their own separate professional organization, the National Association of Colored Graduate Nurses (NACGN) in 1908 after being refused membership in mainstream nursing organizations. Historian Carnegie (2000) asserts that "by organizing, these women proclaimed to the entire profession that they had created an instrument through which they could oppose discrimination in the nursing field on all fronts" (p.93).

Although these grassroots health endeavors fostered a strong sense of racial pride and self-reliance among Black people, it was evident that they could not fully meet the demands of a large and mostly poor Black population who had extensive health needs. Black communities across the country experienced high rates of illness and death, which were attributed by contemporary sources to concentrated poverty, lack of healthcare access, and a scarcity of healthcare providers (Beardsley, 1990; Byrd & Clayton, 2000; DuBois, 2003; Morais, 1967; Smith, 2010). “There are hundreds of communities, indeed, whole counties and parishes in the South, where more than half of the population is colored and there is no public health nurse available, either Negro or white,” nurse activist Estelle Massey Riddle wrote in 1938 (p. 162). At the time, Black nursing schools had only graduated 5,728 nurses to provide care for about 12 million Black people. The growth of the Black nursing workforce was impeded by two interconnected structural barriers: the unequal allocation of resources between Black and white schools, and the dearth of nursing programs accessible to Black women. Although the Supreme Court ruled that equal facilities be provided for Black Americans, municipal and state policymakers ignored this mandate. Most Black women who desired to become registered nurses were educated in underfunded primary and secondary schools that did not provide them the quality of instruction necessary to meet the requirements for admission into nursing school (Carnegie, 1965; Osborne, 1949). Those who did meet the requirements found themselves restricted to a small number of resource-starved nursing schools, where they generally received an inferior education, adversely affecting the institution's reputation and long-term viability. To make clear the extent of the racial disparity in access to educational opportunity, in 1920, there were 40 Black nursing schools in the country in comparison to 1,715 white schools (Hine, 1989).

Furthermore, the growing quest by white nurse leaders to professionalize nursing only further disadvantaged Black nurses. Although Black nurses supported legislative measures to improve and standardize education and practice, they also recognized that, within the constraints of Jim Crow, the enactment of new laws and regulations created more obstacles for their professional advance (Hine, 1989). By the late 1930s, schools in the North that repeatedly failed to fulfill state regulatory criteria lost their credentials, and graduates from these unaccredited schools were not considered for hire by most employers (Anchrum, 2021). The enforcement of stricter educational requirements undoubtedly aided the political objectives of elite white nurses, aiming to establish nursing as a pathway to a better life for poor white women. While their actions were partly inspired by a genuine desire to enhance nursing practice, their hidden motivations were made visible by their deliberate efforts to keep Black women out of nursing organizations, state regulatory bodies, and leadership roles. The political repression of Black nurses mirrored the disenfranchisement of Black voters in the South. The objective was to create significant barriers for Black nurses to exert their influence on nursing and health policies that directly impacted their professional standing and the health of Black Americans (Staupers, 1961). By the onset of World War II, Black nurses recognized the imminent threat to their professional survival. Despite their limited influence within the larger nursing establishment, they would use the organizational power they did possess to wage a strong attack against racial segregation and discrimination.

### World War II

The 1940s witnessed a radical transformation in Black women's approach to enhancing their standing in the nursing profession. Originally centered around the ideals of self-help and economic independence, by the onset of World War II, their strategy had evolved into a vigorous fight for inclusion and equal civil rights. During this time, a now revitalized NACGN emerged as a resounding voice for Black nurses, actively championing progressive nursing and health policy to ensure their demands were heard and influence was felt. The implementation of stricter standards for nursing schools and practice necessitated a more direct approach to combating racial segregation and discrimination (Anchrum, 2021; D’Antonio, 2010). While many schools were forced to close due to their inability to meet higher requirements for operation, the consequences were especially severe for Black nurses. By 1940, the number of Black nursing schools had decreased by 50% from 40 in 1925 to 20, with these schools accounting for 80% of Black student enrollment (Hine, 1989; Staupers, 1961). Moreover, Black nurses represented only 2%, or 8,000, of the total nursing population of approximately 280,500 (Osborne, 1949). With the possibility of war alongside escalating concerns about a nursing shortage, Black nurses saw an opportunity to launch a movement for educational and employment equality. At two crucial junctures in the war, the NACGN, led by President Estelle Massey Riddle and Executive Secretary Mabel Staupers, strategically leveraged the exigencies of war to successfully challenge the discriminatory policies of the federal government and break down racial barriers in nursing.

The NACGN's first battle entailed tackling racial discrimination in federal nurse education programs. With the nation's entry into the war, an urgent need emerged for military nurses. However, removing a quarter of the nurse population for the war effort would severely compromise civilian nursing needs. Therefore, in 1943, the USC implemented a federal nurse education program with the aim of increasing the supply of nurses and maintaining nursing services in the civilian population. The 1943 Bolton Bill established the United States Cadet Nurse Corps (CNC) which allocated $160 million towards the education of nurses and financial assistance for nursing students. Cadet students received free education, a monthly stipend, and uniforms (Roberts, 1954). In the past, the only efforts made on a national scale to recruit Black women into the nursing field were made by the NACGN (Carnegie, 2000). Furthermore, prior federal programs and activities aimed at supporting nurse education were not extended to Black nursing schools (Miller, 1985). This time, Riddle and Staupers took immediate action to ensure that Black schools could benefit from the CNC program. Pressure groups were formed by the NACGN and its supporters to push for the inclusion of an anti-discrimination clause in the Bolton Bill. Thomasina W. Johnson, a lobbyist for the Alpha Kappa Alpha sorority, was recruited to spearhead the campaign. Emphasis was placed on the pressing need to increase the number of nurses and that Black women were an underutilized resource. Johnson's lobbying led to New Jersey Senator Warren W. Barbour introducing an antidiscrimination amendment to the Bolton Bill. Staupers then urged Black nurses to write their representatives and senators and pressure them to support the amendment, while Rita Miller, chair of Dillard University's Division of Nursing, assisted schools in applying for the CNC program (Hine, 1989; Staupers, 1961). Their efforts paid off. In 1945, approximately 2,600 Black students were enrolled in nursing programs, marking the first significant improvement in recruitment of Black nursing students (Staupers, 1961). Moreover, schools of nursing, under pressure to meet the demands of the CNC, extended opportunities to qualified Black applicants. In 1941, only 29 schools, excluding those designated for Black students, were open to Black students. By 1949, the number of schools reporting a non-discriminatory admission policy rose to 354, with Black students actually enrolled in 92 of these institutions (Osborne, 1949). However, this also suggests that, although the bill prohibited racial discrimination for funding eligibility, the vast majority of the 1,225 schools of nursing that participated in the CNC program (1943–1948) did not comply with the requirement, whether in policy or practice (Szecsy, 2016).

The NACGN's second confrontation with the federal government involved challenging the discriminatory practices of the War Department. This battle occurred near the conclusion of the war, in 1945, when Army Surgeon General Norman T. Kirk, in the company of approximately 300 people, announced plans to draft (white) nurses to meet military needs. Staupers quickly pointed out the absurdity of implementing a draft to address the shortage when many qualified Black nurses were being denied the opportunity to serve based on their race. “If nurses are needed so desperately, why isn’t the Army using Negro nurses? Of 9000 registered nurses, the army has taken 247. The navy takes none,” she charged (Hine, 1989, p. 179). War-related industries, like nursing schools, focused solely on the recruitment of young white women. During World War I, Black nurses were nearly barred from military service, and during World War II, they were restricted by racial quotas and limited to caring for Black soldiers and German prisoners of war in segregated military hospitals. War Department officials defended their discriminatory practices by referring to societal norms against race-mixing, the preferences of white soldiers to be cared for by white nurses, and the supposed inferior competence of Black nurses (Threat, 2015). Stauper's decision to challenge Kirk publicly was a deliberate move to bring attention to the War Department's discriminatory policies and their impact on all Americans. She knew that most citizens would oppose the idea of drafting white nurses while many Black nurses were willing to serve but were turned away. Her bold and calculated actions reframed the narrative about the nursing shortage, shifting blame for the shortage from a lack of recruitment of white nurses to the exclusion of Black nurses. Staupers rallied wide support against the draft proposal, mobilizing an interracial coalition of influential community leaders, nurses, women's organizations, political figures, and media voices. Together, they sent letters to the War Department, condemning the unfair treatment of Black nurses. Following widespread public outrage, on January 20, 1945, the War Department conceded and ended racial quotas in the Army Nurse Corps, and 5 days later announced that all eligible nurses would be admitted to the Navy Nurse Corps regardless of color (Hine, 1989; Staupers, 1961).

The NACGN skillfully leveraged the pressures of war to garner support for their movement against racism in nursing, while also exposing the broader structure of medical apartheid to public scrutiny. Their activism illuminated the truth that denying Black Americans their rights also affected the lives of white Americans. Through an organized effort, Black nurses made significant strides in expanding access to nursing education and breaking down racial barriers in employment and professional organizations. At the start of the war, the Army Nurse Corps imposed a quota of 56 Black nurses while the Navy Nurse Corps flatly refused to accept any. By war's end, however, the Army had commissioned over 500 Black nurses, and the Navy had accepted four. Pressures upon the nursing supply also created new job opportunities for Black nurses in the civilian population. As military needs drained white nurses from civilian roles, hospitals and public health agencies turned to Black nurses to meet their staffing demands (Osborne, 1949). Moreover, the War Department's elimination of discriminatory practices, as a staunch defender of Jim Crow law had profound ripple effects. This breakthrough contributed to the gradual dismantling of racial discrimination at the state and local levels, challenging the very ideas that justified segregation. Black nurses' victory over military exclusion also paved the way for the American Nurses Association (ANA) to integrate in 1948, leading to the disbandment of the NACGN three years later (Hine, 1989). This pivotal moment marked, as Carnegie (1965) described, “the end of one era in the fight for equality for all nurses and the beginning of another” (p. 154). Ultimately, their successful challenge to federal discrimination helped lay the foundation for the classical civil rights movement and set the stage for the passage of the Nurse Training Act of 1964.

### The 1950s to 1964

In 1965, nearly two decades after the nursing profession “integrated,” Carnegie queried: “What impact has this integration had on the profession?” (p. 165). According to scholar Hafeeza Anchrum, nursing “integration” — then interpreted as the acceptance of Black women as full members of the ANA followed by the dissolution of the NACGN — had a negative impact on Black nurses' advance. This is because the NACGN was arguably their most powerful tool against white supremacy and discrimination. The disbanding of the organization drastically reduced the momentum of their struggle. There was a marked decrease in the enrollment and graduation of Black nursing students in the 1950s and early 1960s as Black nursing schools continued to close (even as new institutions opened), while the majority of white schools remained inaccessible to Black women (Anchrum, 2021; Carnegie, 2000). Although wartime saw some white nursing programs shift toward more progressive ideals, for most administrators, the true motivation for admitting Black students was the urgent need for nurses. Thus, many of the doors that had previously been open to Black women closed once the war ended (Anchrum, 2021; Osborne, 1949). In 1951, only 236, or 20%, of the 1,170 nursing schools in the nation accepted Black students; and 35 of these schools were exclusively for Black students. Furthermore, the ANA did not assume the responsibility of recruiting Black women into nursing, a role that was once central to the work of the NACGN. Black nursing programs, with few resources, now faced the dual burden of recruiting new students and meeting rising educational standards. Given these challenges, it's likely that the number of Black graduate nurses was also declining (Anchrum, 2021; Carnegie, 1965). Yet, even as the loss of the NACGN weakened their movement in many respects, it did not end it. 

Throughout the 1950s and 1960s, Black nurses continued their fight for inclusion and equality by drawing on their own individual efforts and the support of their medical and educational networks and civil rights organizations (Anchrum, 2021). Nursing schools and hospitals across the nation became battlegrounds for equal rights as Black nurses sought to both access white institutions and preserve those that their communities had created. In Philadelphia, the nurses of Mercy-Douglass Hospital fought to save their nursing program, the only, Black-operated nursing school in the city, following a threat of losing its national accreditation (Anchrum, 2021). A similar struggle emerged at Meharry Medical College School of Nursing in Nashville (Carnegie, 1964). Fed up with injustice but steadfast in their quest for freedom, Black nurses increasingly sought assistance from civil rights organizations. For example, Esther Cready, supported by the National Association for the Advancement of Colored People, filed a successful lawsuit against the University of Maryland School of Nursing after being denied admission due to her race. In 1953, she became the school's first Black graduate. Her case helped lay the foundation for the landmark 1954 Supreme Court decision in *Brown v. Board of Education*, which declared racial segregation in public schools unconstitutional (Maryland State Archives, 2020). Local civil rights groups also played a key role in supporting Black nurses as they gradually integrated the staffs of white hospitals, where they faced racially hostile environments and unfair treatment. At a Catholic hospital in West Virginia, for example, twenty white nurses and several doctors walked off the job after three Black nurses were hired. Although their opportunities for hospital work were limited, some Black nurses also quit their jobs in protest of racial indignities such as being forced to eat in a separate dining hall (Anchrum, 2021). Complaints of workplace discrimination from healthcare staff were echoed by Black patients and community groups, who increasingly filed grievances with local civil rights organizations about unequal medical treatment (Byrd & Clayton, 2000; McBride, 1989). Black nurses also participated in broader civil rights efforts. They supported school desegregation in their communities, joined the 1963 March on Washington for Jobs and Freedom, and in 1964, served on a human rights committee providing medical care to civil rights activists in Mississippi (Anchrum, 2021; Dittmer, 2009; Fee et al., 2002).

It was within this context of social revolution, growing national concerns over health personnel shortages, and increased advocacy from activists, nurses, and hospital leaders, the USC enacted the Nurse Training Act (NTA) on September 4, 1964. President Johnson described it as “the most significant nursing legislation in the history of our country” (Rubenfeld et al., 1981, p. 1202). The NTA allocated $287.6 million over five years to improve the quantity and quality of nursing care through four key initiatives: construction grants for nursing schools, special project grants to strengthen the quality of training, loans for students and schools, and expansion of the traineeship program for advanced education (United States Public Health Service, U.S. Department of Health, Education, and Welfare, Public Health Service, Division of Nursing, 1965). The NTA was enacted in response to the report, “Toward Quality in Nursing,” issued by the Surgeon General's Consultant Group on Nursing. The report identified long-standing issues driving the nursing shortage, including declining student enrollments, recruitment challenges, an inadequate educational system, low wages, and poor working conditions (United States Public Health Service, U.S. Department of Health, Education, and Welfare, 1963). These issues were even more severe for Black nurses, who faced lower pay, harsher working conditions, and greater obstacles in recruitment compared to their white counterparts. Moreover, while the 1960s saw some racial progress — such as the opening of five new Black nursing schools and a rise in white nursing schools purporting non-discriminatory admission policies — significant inequalities persisted. A 1963 survey revealed that of the 82% of schools with reported policies to admit Black students, less than half actually enrolled them (Carnegie, 1965). The NTA aimed to address these disparities.

The federal government not only funded nursing education but also removed a major barrier inhibiting the expansion of the nursing workforce by eradicating racial segregation in professional nursing schools. Unlike the anti-discrimination provision in the Bolton Bill of WWII, which allowed federal funding for segregated schools and thus limited Black Americans' access to program benefits, the NTA was governed by Title VI of the Civil Rights Act of 1964, passed just two months earlier. Title VI prohibits discrimination based on race, color, or national origin in all federally funded programs (USC, 1964; United States Public Health Service, U.S. Department of Health, Education, and Welfare, Public Health Service, Division of Nursing, 1965). As a result, nursing schools that excluded or discriminated against Black Americans were disqualified from receiving NTA funds. In 1965, Carnegie noted the impact of this legislation on nursing school integration: “If for no other reason than to qualify for federal funds, many schools that had previously excluded Negro students are now making a concerted effort to find Negro students to apply for admission” (p. 155). To monitor and enforce these standards, the NTA established the National Advisory Council on Nurse Training, tasked with reviewing applications and recommending funding to the Surgeon General. Additionally, the Public Health Service's Division of Nursing established a Nursing and Education Training Branch, which provided policy guidance, assisted with application preparation, and conducted site visits to ensure that schools met all requirements (Miller, 1985; United States Public Health Service, U.S. Department of Health, Education, and Welfare, Division of Nursing, Program Review Committee, 1967). 

Desegregating nursing schools, however, did not guarantee a rise in the number of Black students enrolled. To see a substantial increase in the population of Black nurses required time and coordinated efforts from both the nursing profession and the federal government. For decades, white nursing schools made little to no effort to recruit Black students, often justifying their exclusion by claiming Black applicants were unqualified. This was no longer a sufficient rationale. Under the NTA, schools seeking federal funds were now required to create action plans to integrate their student bodies (Carnegie, 1965). Subsequent amendments to the NTA introduced special project funds to support schools in this effort and address the unique needs of minority students. The funds were used to establish “bridge for entry into nursing” programs, provide remedial and tutoring services, and allocate resources for recruitment to identify potential minority students. These initiatives aimed to help students gain admission to nursing programs and succeed academically. The anticipated impact of desegregating public schools following *Brown v. Board of Education* was expected to further improve Black students' educational preparation. The NTA Student Loan Program also provided critical support, making nursing education more accessible to Black students who could not afford the cost of tuition. Black nursing programs, which had long struggled with underfunding, were finally eligible to apply for federal assistance to enhance and expand their facilities and programs (United States Public Health Service, U.S. Department of Health, Education, and Welfare, Division of Nursing, Program Review Committee, 1967). Additional legislation furthered supported these efforts. The Higher Education Act of 1965 expanded financial aid for low-income students and provided institutions with funds for special admission and support programs for minority students (Carnegie, 2000). The federal government also leveraged funds from the Medicare Act of 1965 to force the desegregation of hospitals, essentially using the same approach to that applied in nursing schools (Smith, 2016). This requirement desegregated the few remaining race-restrictive hospital nursing schools. Together, these federal policies influenced state and local governments to advance racial integration, strengthening the infrastructure for a more equitable system of healthcare provision (Carnegie, 2000).

## Conclusion

The 1964 implementation of the NTA marked a pivotal moment in Black nurses’ decades-long struggle for equal access to education and federal support. The Act's desegregation requirements and accompanying provisions opened doors to educational opportunities that had long been closed to Black people aspiring to become registered nurses, thereby contributing to the evolution of the nursing workforce into the racially, ethnically, gender and class diverse entity it is today. By 2022, Black nurses constituted 13% of the nursing workforce, a dramatic increase from the 2% recorded in 1950 (American Association of Colleges of Nursing, 2024). For these reasons, the NTA stands among the era's most progressive legislation, supporting racial and healthcare equality and reinforcing the Civil Rights Act of 1964.

Viewing this legislation through the eyes of Black nurses reveals not only its historical significance but also valuable lessons for tackling issues pertaining to health inequity today. It challenges us to reflect on the government's role in protecting the well-being and rights of its citizens, on the power and sustainability of progressive policies, and on the ongoing need for organized resistance against institutionalized oppression. This is especially critical now, as political challenges to affirmative action and diversity, equity and inclusion programs threaten to undo gains made by the civil rights movement (Blackstock et al., 2024). Reversing these initiatives could further strain an already depleted nursing workforce, as many experienced nurses approach retirement and the healthcare needs of an aging population rise (American Association of Colleges of Nursing, 2024). Furthermore, these shifts would have an outsized impact on Black Americans, who, due to the legacies of slavery and Jim Crow, continue to face high rates of morbidity and mortality, persistent poverty, and disparities in healthcare access and education. Undoing progress in racial equity policies and initiatives would deepen these injustices, underscoring why continued movement work is essential to protect the rights of society's most vulnerable.

The struggle of Black nurses teaches us that we cannot depend solely on the ruling class to do what is righteous and just. We must hold onto our self-determination while also holding those in power accountable. This message resounded in 1908 when Black nurses organized the NACGN, and again in 1971, when they reconvened to form the National Black Nurses Association (NBNA) under Dr. Lauranne Sams, in response to the ANA’s failure to effectively represent their needs and interests (Carnegie, 2000). The NBNA continues the NACGN's legacy, striving to combat racial discrimination in nursing and advocating for health equity. With over a hundred chapters, the organization provides programs focused on education, public health, mentoring, and health policy. Yet, as Black nurse leaders declared in 1950, the fight for social justice is a call to action for every nurse. It requires a collective commitment to confront and eradicate anti-Black racism deeply ingrained in the nation's fabric. It demands a critical examination and understanding of the past in order to create a different future—recognizing that every step toward a just and humane society has been hard won, not freely given. And the Nurse Training Act of 1964 was no exception.

## Notes

1. Capitalizing the “B” in “Black” signifies the reclamation of the humanity, dignity, and agency of people of African descent, who have historically endured dehumanization on the basis of their skin color. At the turn of the twentieth century, sociologist W.E.B. Du Bois protested the use of a lowercase “n” in “Negro” in reference to people of the African diaspora. He considered “the use of a small letter for the name of twelve million Americans and two hundred million human beings a personal insult” as other racial groups were referenced with uppercase letters. Moreover, capitalizing the term “white” continues to be a convention among white supremacists, and adhering to this practice may inadvertently validate such ideologies. For more discussion on this topic see, Constance Backhouse, Colour-Coded: A Legal History of Racism in Canada, 1900-1950 (University of Toronto Press, 1999), pp. 1–17 and Lori L. Tharps (2014). The case for Black with a capital B. *New York Times*.
